# COVID-19 Pneumonia Diagnosis Using Medical Images: Deep Learning–Based Transfer Learning Approach

**DOI:** 10.2196/75015

**Published:** 2025-09-26

**Authors:** Anjali Dharmik

**Affiliations:** 1 Royal Holloway University of London, Egham Hill, Egham, TW20 0EX, United Kingdom, 44 7867304854

**Keywords:** computer vision, COVID-19 pneumonia diagnosis, deep learning, transfer learning, medical imaging analysis

## Abstract

**Background:**

SARS-CoV-2, the causative agent of COVID-19, remains a global health concern due to its high transmissibility and evolving variants. Although vaccination efforts and therapeutic advancements have mitigated disease severity, emerging mutations continue to challenge diagnostics and containment strategies. As of mid-February 2025, global test positivity has risen to 11%, marking the highest level in over 6 months, despite widespread immunization efforts. Newer variants demonstrate enhanced host cell binding, increasing both infectivity and diagnostic complexity.

**Objective:**

This study aimed to evaluate the effectiveness of deep transfer learning in delivering a rapid, accurate, and mutation-resilient COVID-19 diagnosis from medical imaging, with a focus on scalability and accessibility.

**Methods:**

An automated detection system was developed using state-of-the-art convolutional neural networks, including VGG16 (Visual Geometry Group network-16 layers), ResNet50 (residual network-50 layers), ConvNeXtTiny (convolutional next-tiny), MobileNet (mobile network), NASNetMobile (neural architecture search network-mobile version), and DenseNet121 (densely connected convolutional network-121 layers), to detect COVID-19 from chest X-ray and computed tomography (CT) images.

**Results:**

Among all the models evaluated, DenseNet121 emerged as the best-performing architecture for COVID-19 diagnosis using X-ray and CT images. It achieved an impressive accuracy of 98%, with a precision of 96.9%, a recall of 98.9%, an *F*_1_-score of 97.9%, and an area under the curve score of 99.8%, indicating a high degree of consistency and reliability in detecting both positive and negative cases. The confusion matrix showed minimal false positives and false negatives, underscoring the model’s robustness in real-world diagnostic scenarios. Given its performance, DenseNet121 is a strong candidate for deployment in clinical settings and serves as a benchmark for future improvements in artificial intelligence–assisted diagnostic tools.

**Conclusions:**

The study results underscore the potential of artificial intelligence–powered diagnostics in supporting early detection and global pandemic response. With careful optimization, deep learning models can address critical gaps in testing, particularly in settings constrained by limited resources or emerging variants.

## Introduction

### Background

SARS-CoV-2, the virus responsible for COVID-19, first emerged on December 31, 2019, in Wuhan City, Hubei Province, China [1]. It is a highly transmissible respiratory pathogen capable of causing severe illness or death across all age groups [2]. Since its initial outbreak, substantial progress has been made in managing the virus through vaccination, antiviral therapies, and diagnostic technologies powered by artificial intelligence (AI).

Despite these advances, SARS-CoV-2 continues to pose a global health challenge, especially for immunocompromised individuals and those with underlying conditions. One of the most persistent obstacles is the virus’s ability to mutate rapidly. To date, more than 26 genetically distinct variants have been identified, many of which exhibit increased transmissibility and immune evasion due to mutations that enhance their binding affinity to host cells [3].

By August 20, 2023, the pandemic had resulted in over 769 million confirmed cases and more than 6.9 million deaths worldwide [4]. Early in the pandemic (January 30, 2020), the World Health Organization (WHO) declared COVID-19 a public health emergency of international concern [5].

More recently, SARS-CoV-2 has shown a global resurgence. As of May 11, 2025, surveillance data from the Global Influenza Surveillance and Response System indicated that the global test positivity rate reached 11%, up significantly from 2% in February 2025 [6]. This current wave, comparable to the July 2024 peak of 12%, is largely driven by cases in the Eastern Mediterranean, South-East Asia, and the Western Pacific Region [6].

A key driver of this resurgence is the emergence of the recombinant XEC variant, first detected in Germany in June 2024 [7]. Derived from the 2 Omicron subvariants KS.1.1 and KP.3.3, XEC rapidly spread worldwide, and by December 2024, it accounted for nearly 45% of cases in the United States [3,7-9]. Its global dominance underscores the critical importance of continued genomic surveillance and adaptive diagnostic strategies.

In February 2025, the WHO categorized circulating variants as follows: dominant variant: XEC; variant of interest: JN.1 (known for partial immune evasion) [10]; variants under monitoring: KP.2, KP.3, KP.3.1.1, JN.1.18, LB.1, XEC, and LP.8.1 (potential impact on transmission and immunity) [10]. Compared to January 2024, when variants like EG.5 (Eris) and FL.1.5.1 (Fornax) dominated, the landscape has shifted greatly in 2025, with XEC and JN.1 overtaking earlier subvariants such as XBB.1.16 (Arcturus) [3]. The evolution of COVID-19 variants and their global impacts are presented in Table 1.

**Table 1.** Evolution of dominant COVID-19 variants and their global impact (January 2024-February 2025).

Time period	Dominant/high-prevalence variants	Key characteristics	Status by February 2025
January 2024	EG.5 (Eris): 24.5% FL.1.5.1 (Fornax): 13.7% XBB.1.16 (Arcturus): declining presence	Derived from Omicron lineages Moderate immune escape	Largely replaced by newer variants
July 2024	Mixed circulation; early rise of XEC	XEC began spreading in Europe	Became dominant by late 2024
December 2024	XEC 45% in the United States Increasing in Europe and Australia	Recombinant of KS.1.1 + KP.3.3 High transmissibility	Global spread accelerating
February 2025	XEC: dominant globally JN.1: variant of concern Variants under monitoring: KP.2, KP.3, LP.8.1, etc	Enhanced immune evasion Multiple regions affected	Driving the recent case surge

### Symptoms

COVID-19, caused by the SARS-CoV-2 virus, primarily affects the respiratory system, with symptoms ranging from mild upper respiratory issues to severe lung involvement. While most cases are mild, individuals with comorbidities (cardiovascular disease, diabetes, or cancer) are at higher risk for complications [11].

Variants like Delta have shown a preference for the lower respiratory tract, leading to lung consolidation and pneumonia, which are features identifiable on computed tomography (CT) scans and X-rays. In contrast, Omicron subvariants tend to affect the upper airways more, often resulting in less severe radiological findings [12]. However, symptomatology continues to evolve with emerging variants, influencing the type and severity of pulmonary involvement seen in medical images [3]. The correlations between clinical symptoms and radiological patterns are presented in Table 2.

**Table 2.** Correlation between clinical symptoms and radiological patterns in COVID-19 diagnosis.

Symptom	Radiological pattern	Imaging modality	Relevance to the study
Dry cough	GGOs^a^, peripheral opacities	CT^b^, X-ray	Frequently observed in mild to moderate COVID-19 pneumonia
Shortness of breath	Bilateral GGOs, interstitial thickening	CT, X-ray	Indicates lower lung involvement; key pattern for classification
Fever	Often present alongside GGOs	CT	Supports image-based diagnosis when combined with lung findings
Hypoxia	Diffuse alveolar damage, ARDS^c^-like patterns	CT	Seen in severe cases; helps the model identify critical patterns
Chest pain	Subpleural consolidations, patchy opacities	CT	May reflect inflammatory involvement; assists in differentiation
Long COVID symptoms	Fibrotic changes, residual GGOs	CT	Useful for tracking persistent lung changes in follow-up scans

a GGOs: ground-glass opacities.

b CT: computed tomography.

c ARDS: acute respiratory distress syndrome.

### Related Work

In response to the global impact of COVID-19, a wide range of clinical and technological strategies have been developed to support diagnosis, treatment, and containment. Among these, imaging-based AI systems have emerged as promising tools for timely and accessible COVID-19 diagnosis, particularly in resource-limited and high-burden settings. However, a review of the existing literature revealed notable challenges in data diversity, standardization, and model generalizability.

#### Telehealth Services

The rapid expansion of telemedicine platforms enabled remote assessment and monitoring of COVID-19 patients, especially during peak transmission periods when hospital resources were overwhelmed [13]. However, telehealth often lacks the diagnostic depth provided by imaging or laboratory testing and is generally used for symptom tracking and triage rather than precise diagnosis.

#### Imaging-Based Diagnostics

Chest X-rays and CT scans have been instrumental in identifying characteristic COVID-19 lung involvement, including bilateral ground-glass opacities and consolidations [14]. Numerous deep learning models have been developed for pneumonia and COVID-19 detection using chest X-ray and CT data. For example, MobileNet (mobile network) achieved 94.2% and 93.7% accuracy on 2 public chest X-ray datasets containing 5856 and 112,120 images, respectively [15]. Despite these benefits, existing studies often suffer from limited and nonstandardized datasets, a lack of demographic metadata (age and sex), and geographical imbalance, reducing generalizability. In a separate study using InceptionV3 and convolutional neural network (CNN) models on a Kaggle X-ray dataset of 7750 images, the researchers reported impressive results (accuracy: 99.2%, recall: 99.7%) [16]. However, the use of a single public dataset lacking demographic diversity and external validation limits generalizability.

A CT-based study using NASNet achieved an exceptionally high accuracy of 99.6%, with a sensitivity of 99.9% and a specificity of 98.6% [17]. However, this evaluation was based on a small, imbalanced dataset of 249 patients, with no external validation, no interpretability tools, and no metadata analysis (eg, age, sex, and geography), weakening its clinical reliability and fairness. Furthermore, alternative architectures like ResNet or VGG were not benchmarked, and hyperparameter tuning was minimally discussed.

These limitations underscore the need for scalable, diverse, and metadata-rich imaging datasets to enhance model reliability and cross-population performance.

#### Diagnostic Technologies: Strengths and Limitations

While reverse transcription–quantitative polymerase chain reaction (RT-PCR) remains the diagnostic gold standard [18], its accuracy can be impacted by emerging variants and sample quality. In response, several alternative diagnostic technologies have been explored. A comparison of key methods is presented in Table 3.

**Table 3.** Comparative overview of diagnostic techniques.

Method	Advantages	Limitations
Mutation-specific/multiplex PCR^a^	High sensitivity (98.6%) and multiplex variant detection	Requires prior mutation knowledge
Loop-mediated amplification	Fast, simple, ≥90% sensitivity, and suitable for low-resource settings	Prone to false positives and less stable
CRISPR-Cas detection	100% specificity, cost-effective, rapid, and suitable for POC^b^ use	Low sensitivity at low viral loads (53.9%) and detects only point mutations
RT-PCR^c^	Precise quantification and highly sensitive	Expensive and complex instrumentation
Rapid antigen test	Quick, user-friendly, low-cost, and suitable for self-testing	Lower sensitivity and affected by viral load and sample collection
ELISA^d^	High throughput, useful for antibody screening, and suitable for POC use	Variant-driven antigenic drift affects sensitivity
Lateral flow assay	Home use–friendly and long shelf-life	Detects limited antigenic sites and lower sensitivity
Viral genome sequencing	Enables variant tracking and mutation identification	Time-consuming, costly, and resource-intensive

a PCR: polymerase chain reaction.

b POC: point-of-care.

c RT-PCR: reverse transcription–quantitative polymerase chain reaction.

d ELISA: enzyme-linked immunosorbent assay.

PCR-based methods are highly accurate but not variant-agnostic. Antigen-based tests are accessible but less reliable. Genome sequencing is ideal for surveillance but not rapid diagnosis. These constraints further support the need for AI-powered imaging diagnostics that are scalable, noninvasive, and rapid.

#### Imaging-Based Deep Learning as a Complementary Tool

Deep learning applied to medical imaging presents a promising complementary diagnostic method, particularly in areas with limited laboratory capacity. Yet, current research has notable limitations. For instance, a protocol paper of a prospective AI model for chest X-ray images highlights the intention to use 600 images [19]. However, it lacks clear details on geographic and demographic diversity, metadata tracking (eg, age and sex), and model architecture. Moreover, it does not describe how biases will be addressed or how low-prevalence conditions will be handled, which can be considered critical for real-world implementation.

Given the diagnostic delays and limitations associated with conventional methods, deep learning applied to medical imaging offers a promising complementary approach. Models trained on chest X-rays and CT scans can provide rapid, accurate, and interpretable results, which are particularly critical in settings where molecular testing is delayed or inaccessible. In this study, these efforts were built upon by employing transfer learning on an expanded, standardized imaging dataset to enhance diagnostic accuracy and generalizability. This approach addresses prior limitations related to data volume, diversity, and model robustness.

### Challenges

Despite substantial progress since 2020, several evolving challenges continue to hinder reliable COVID-19 detection, particularly due to viral mutations, overlapping disease presentations, and infrastructural limitations.

#### Emerging Variants Reduce Test Sensitivity

New SARS-CoV-2 variants, such as Pi, Rho, XEC, and JN.1, exhibit mutations in the spike (S) and nucleocapsid (N) proteins, which impair molecular and antigen-based diagnostic assays [20]. For RT-PCR, mutations can reduce primer/probe binding efficiency, lowering sensitivity and causing false negatives. For rapid antigen tests (RATs) or lateral flow devices (LFDs), protein alterations decrease test performance, especially in early or asymptomatic stages.

#### Diagnostic Overlap in Imaging

Radiological signs of COVID-19 (ground-glass opacities) overlap with other pulmonary infections, including bacterial pneumonia, influenza, tuberculosis, respiratory syncytial virus, and fungal infections. This nonspecificity complicates diagnosis, especially without clinical or laboratory correlation, increasing the risk of false positives or misclassification.

#### Dataset Limitations in AI-Based Diagnosis

Many existing AI models are trained on limited or biased datasets, which can impact their generalizability. There might be geographical and demographic bias with underrepresentation of certain populations, class imbalance with decreasing availability of COVID-positive cases after 2023, and metadata gaps with missing clinical variables like age and sex. These limitations reduce model robustness, especially in real-world settings with varied patient populations.

#### Barriers to Clinical AI Integration

Despite promising research, AI tools face challenges in clinical adoption, including a lack of regulatory validation (Food and Drug Administration approval/Conformité Européenne certification), poor integration with electronic health records (EHRs), and clinician skepticism due to a lack of explainability or interpretability. Without improved trust, transparency, and workflow compatibility, real-world deployment remains limited.

#### Data Privacy and Collaboration Constraints

Privacy regulations (Health Insurance Portability and Accountability Act and General Data Protection Regulation) and institutional data silos restrict access to multicenter, diverse datasets and large-scale, cross-border collaborations necessary for robust AI development.

#### Reinfections and Long COVID Monitoring

Most diagnostic tools are optimized for acute-phase detection. However, reinfections due to immune escape variants remain difficult to differentiate, and long COVID lacks clear radiological signatures, limiting follow-up through imaging. There is a need for diagnostic systems that can also support longitudinal patient monitoring.

#### Infrastructure Limitations in Resource-Constrained Settings

Low-income regions often lack access to RT-PCR labs, CT or X-ray imaging facilities, and high-performance computing resources for AI deployment. This exacerbates health inequities and delays early detection and containment efforts.

### Solution

This study presents a transfer learning–based deep learning framework for the accurate and mutation-resilient diagnosis of COVID-19 using chest radiological imaging (X-rays and CT scans). The approach addresses limitations in conventional diagnostics.

#### Mutation-Resilient Design

Unlike RT-PCR and antigen tests that rely on viral RNA or surface protein stability, the present image-based approach detects disease-induced radiological changes, remaining unaffected by emerging variants or antigenic drift.

Imaging-based models do not depend on spike or nucleocapsid protein integrity, making them robust against variants like XEC and JN.1.

#### Advanced Transfer Learning Architecture

Transfer learning has been adopted using pretrained CNNs on ImageNet, and they have been fine-tuned on curated COVID-19 datasets with advanced preprocessing, augmentation, and optimization strategies.

#### Fine-Grained Classification

The system is designed for binary classification (COVID-19 vs normal) and multiclass classification (COVID-19 pneumonia vs non-COVID pneumonia vs normal), depending on available label granularity. Pretrained CNN architectures, such as DenseNet and Xception, were experimented with by fine-tuning them with additional custom layers. The models were further optimized through hyperparameter tuning, and attention modules were incorporated to improve the network’s ability to focus on COVID-relevant regions in the lung fields.

#### Diverse, Multiregional Dataset

To improve generalization, a dataset of 25,195 labeled images has been assembled across CT and X-ray modalities; multiple regions (Asia, Europe, and North America); and varying age groups, ethnicities, and imaging protocols. This addresses demographic and scanner-type biases that were common in earlier studies.

#### Interpretability and Clinical Integration

Grad-CAM visualizations have been integrated for transparent decision support.

#### Longitudinal Monitoring Capabilities

The present framework has been designed to be extended for follow-up analysis, allowing radiological tracking of postinfection abnormalities and aiding in long COVID assessment and reinfection detection.

#### Edge and Cloud Deployment Readiness

The final model has been compressed using quantization and pruning techniques for deployment in edge devices (mobile apps and local hospital servers) and cloud-assisted diagnostic platforms.

### Motivation

Despite a global decline in COVID-19 mortality by March 2025, accurate and rapid diagnosis remains essential due to the continued emergence of novel SARS-CoV-2 variants and the absence of a universal treatment [11]. Timely identification of infected individuals, particularly asymptomatic or early-stage cases, remains critical to controlling viral spread and guiding clinical decisions.

#### Limitations of Conventional Diagnostic Methods

Traditional approaches like RT-PCR, LFDs, and RATs, though widely used, suffer from several drawbacks: reduced sensitivity with emerging variants due to mutations in target genes and proteins; delayed turnaround times in lab-based settings; sample quality dependency leading to false negatives, especially in asymptomatic individuals; and lower reliability in detecting newer variants such as Pi, Rho, XEC, and JN.1. These limitations necessitate complementary, mutation-resilient diagnostic strategies.

#### Potential of Medical Imaging

Chest CT scans and X-rays have proven valuable in identifying COVID-19–induced pneumonia, with CT offering higher sensitivity (88%‐97%) and X-rays being cost-effective and more widely available, especially in resource-constrained environments [4].

The application of deep learning and transfer learning to radiological image analysis enhances diagnostic accuracy, speed, and consistency, independent of viral genome variability or test kit supply chains.

#### Study Objectives

This study developed and evaluated a deep learning diagnostic framework using CT and X-ray images to detect COVID-19 pneumonia. The key goals were to achieve a diagnostic accuracy of >95% across multiple viral variants; improve generalization across populations, regions, and imaging devices; differentiate COVID-19 pneumonia from other respiratory conditions with overlapping features; and benchmark the model’s performance against traditional diagnostic methods.

#### Radiological Overlap With Other Pulmonary Conditions

To ensure clinical reliability, the model must distinguish COVID-19 pneumonia from visually similar conditions. The radiological overlap emphasizes the need for fine-grained classification models capable of accurately distinguishing COVID-19 from similar pulmonary pathologies using feature-rich image interpretation.

This study aimed to develop a mutation-resilient deep learning framework for accurate COVID-19 diagnosis using CT and X-ray imaging, overcoming challenges faced by traditional RT-PCR and antigen tests due to emerging SARS-CoV-2 variants. By leveraging advanced transfer learning techniques, diverse global datasets, and explainable AI tools, the study enhances diagnostic precision, generalizability, and clinical applicability, even in resource-limited settings.

## Methods

### Research Questions

This study investigated the viability of transfer learning–based deep learning approaches for COVID-19 pneumonia detection using CT and X-ray imaging. It specifically explored the following areas:

1. Diagnostic accuracy: Can a transfer learning–based deep learning model accurately diagnose COVID-19 pneumonia, including cases caused by emerging variants (Pi, Rho, Xec, and JN.1), using CT and X-ray images?
2. Comparative diagnostic performance: How does the model’s performance compare to conventional diagnostic methods, such as RT-PCR, LFDs, and RATs, particularly in the presence of viral mutations?
3. Generalizability across populations and regions: Does training on a diverse, multiregional, and multivariant dataset improve the generalizability and robustness of the deep learning model?
4. Differentiation from other pneumonias: Can the proposed model effectively distinguish COVID-19 pneumonia from non-COVID pneumonia conditions using imaging data?

### Data Collection

To address the research questions, a large-scale dataset was curated by aggregating CT and X-ray images from publicly available, ethically approved sources, ensuring inclusion across age groups, genders, countries, and COVID-19 variants.

#### Source Overview

The dataset comprised radiological data from 9 primary sources. Each source was selected based on the following inclusion criteria: confirmed diagnostic status, with only RT-PCR–confirmed COVID-19 cases and clinically validated normal or pneumonia samples included; radiological quality, with DICOM or high-resolution image formats (PNG and JPEG) and clear lung visibility; and metadata completeness, with availability of patient demographics (age and sex), scan modality, and clinical context, where applicable.

#### Summary of Collected Imaging Datasets

The following imaging datasets were considered:

1. Lung Image Database Consortium image collection (LIDC-IDRI) [21] (United States): A well-known X-ray dataset primarily used for lung nodule detection and normal case baselines
2. Società Italiana di Radiologia Medica e Interventistica (SIRM) [22] (Italy): Collection of chest X-ray images from confirmed COVID-19 patients shared by the Italian Society of Medical and Interventional Radiology
3. Banco de Imágenes Médicas de la Comunidad Valenciana–COVID-19 (BIMCV-COVID19) [23] (Spain): Comprehensive dataset containing both CT and X-ray images with annotated severity scores and clinical metadata
4. China National Center for Bioinformation (CNCB; normal) and CT images and clinical features for COVID-19 (iCTCF; COVID) [24] (China): Paired datasets offering CT and X-ray scans from healthy subjects (CNCB) and confirmed COVID-19 cases (iCTCF)
5. The Cancer Imaging Archive (TCIA) [25] (United States): CT images from TCIA, used to supplement lung imaging studies
6. Medical Imaging Data Resource Center - RSNA International COVID-19 Open Radiology Database (MIDRC-RICORD) series (United States):
  - RICORD-1A [26]: COVID-19 CT scans with expert annotations
  - RICORD-1B [27]: Normal CT images for balanced model training
  - RICORD-1C [28]: Additional COVID-19 scans to expand diagnostic variety
7. Study of Thoracic CT in COVID-19 (STOIC) [29] (France): Over 2000 annotated CT scans from a national COVID-19 detection program
8. Radiopaedia [30] (global): Open-access repository of CT and X-ray images contributed by medical professionals worldwide
9. MosMedData [31] (Russia): CT scans of COVID-19 patients categorized by severity, including mild, moderate, and severe cases

### Data Preprocessing

The dataset, while large and geographically diverse, presents a notable class imbalance, primarily due to the disproportionate contribution from the BIMCV-COVID19 collection (Spain) [23]. COVID-19–positive cases (59,961) significantly outnumber normal and non-COVID pneumonia cases (27,270). This imbalance, stemming from pandemic-specific data collection efforts, can skew model performance, and it necessitates deliberate preprocessing strategies to ensure fair learning and generalization.

#### Addressing Class and Source Imbalance

To correct for imbalance and ensure representative learning, undersampling of Spain was performed to reduce overrepresentation, and countries with fewer than 100 total samples were removed to prevent noise and overfitting.

#### Handling Missing Data

Significant missing values were found in the metadata. Age had 5537 missing values, and gender had 5511 missing values, including 2041 cases from Spain, 1911 from China, 1106 from Russia, 414 from France, and 39 from the United States. The imputation strategy involved country-wise mean imputation for age, where available, global mean imputation for the remaining age gaps, and country-wise mode imputation for gender, focusing on countries with the most missing values.

#### Age Outliers and Grouping

The age range was 0 to 100 years. Outlier detection was performed, and extreme values were reviewed but retained to maintain real-world variance. Patients were categorized into discrete age groups (eg, 0‐18, 19‐35, 36‐60, and 61+ years), allowing demographic stratification during training. To handle age group imbalance during dataset splitting, the stratify label *by age group* was applied.

#### Data Filtering and Preparation

The number of final images after metadata curation was 11,052 (8842 for training and 2210 for validation). The preprocessing pipeline included image resizing to 75×75 pixels with 3 channels (RGB) and normalization with pixel values rescaled to [0, 1].

#### Country-Level Label Distribution

The distribution of COVID-19 and normal images is presented in Multimedia Appendix 1. Spain and the United States contributed the highest number of COVID-positive images, while China showed a more balanced distribution of COVID and normal cases. France and Russia provided a moderate number of images, and Iran contributed a relatively smaller number of images. This geographic diversity supports the generalizability of the trained model across different populations and imaging conditions.

#### Data Augmentation for Country-Level Balancing

To balance samples across underrepresented countries, the following augmentation techniques were applied: random horizontal flip, random rotation (15°), random zoom (10%), random contrast (10%), and random translation (5%).

#### Category-Level Augmentation

Despite country-level augmentation, class imbalance between the COVID-19 and normal categories persisted. Additional category-level augmentation was applied to underrepresented normal samples to achieve closer class parity, helping reduce bias during model training.

### Modeling

#### Dataset Overview

After applying data augmentation techniques, the final dataset consisted of 24,408 medical images, which were stratified to maintain balanced class distributions across all subsets. The dataset was divided into 19,527 images for training, 4881 for validation, and 952 for testing. Stratified sampling ensured proportional representation of each class, supporting fair evaluation and reducing potential bias during model training and validation.

#### Data Preprocessing

All images were resized to 224×224 pixels to ensure consistent input dimensions compatible with standard CNNs. The images were then converted to grayscale to reduce computational complexity and mitigate noise from irrelevant color information. Pixel intensities were normalized to stabilize training dynamics.

To determine an optimal batch size for training, an analysis was performed regarding how different batch sizes divide the total training dataset of 19,527 records. This involved calculating how many steps (batches) each epoch would require for various batch sizes. Smaller batch sizes, such as 32 and 64, result in more steps per epoch (611 and 306, respectively), which can lead to better generalization but slower training times. On the other hand, very large batch sizes like 512 or 1024 reduce the number of steps significantly but may hinder model generalization and require careful tuning of the learning rate. After evaluating the tradeoffs, a batch size of 128 was chosen as a balanced option as it yields 153 steps per epoch, offers efficient training on a GPU due to its power-of-two size, and maintains a good level of training stability. This choice reflects a compromise between computational efficiency and model performance, ensuring the training process remains both practical and effective.

To address class imbalance, a combination of data augmentation and undersampling strategies was implemented. The dataset was split into 80% for training and 20% for validation, and performance was further optimized using caching and shuffling for the training set. For the validation set, caching alone was applied to ensure consistent evaluation.

To enhance the randomness of the training data, the buffer size was set to 10,000 during the shuffling process. The buffer size determines how many samples are held in memory and randomly shuffled at any given time before being passed to the model in batches. A smaller buffer size, such as 100 or 1000, can result in less effective shuffling, especially with larger datasets, as only a limited portion of the data is randomly sampled at a time. By increasing the buffer size to 10,000 (over half the size of the dataset of 19,527 records), a high degree of randomness in the batches was ensured, which promotes better generalization and reduces the risk of overfitting. Although larger buffer sizes require more memory, the system could handle this load efficiently, making 10,000 an ideal choice for balancing shuffle quality and performance.

#### Model Architecture

A structured and modular deep learning pipeline was developed for hyperparameter optimization and fine-tuning using TensorFlow and Keras Tuner. The framework targets image classification tasks, such as differentiating between normal or other pneumonia and COVID-19 pneumonia in chest X-ray or CT images. The pipeline combines automated hyperparameter tuning, transfer learning, and robust training strategies to improve classification accuracy and generalization, which are particularly crucial when dealing with limited medical datasets.

The model was trained over 30 epochs with a batch size of 128, a buffer size of 10,000, and a fixed random seed of 42 to ensure reproducibility.

To determine the optimal number of training epochs without overfitting, early stopping was used, which is a regularization technique that monitors validation performance during training. Instead of predefining a fixed number of epochs, early stopping halts training once the validation loss stops improving for a set number of consecutive epochs (patience). This dynamic approach allows the model to train just long enough to reach optimal performance without wasting computation or risking overfitting. Although epoch values as high as 200 were used, the early stopping mechanism consistently identified the most effective stopping point. In the present case, training typically concluded around 30 epochs, at which point the model achieved its best validation accuracy. This method provided an efficient and reliable way to control training duration while ensuring strong generalization.

At the core of the architecture was a transfer learning model based on VGG16 (Visual Geometry Group network-16 layers), which was selected as the baseline due to its simple, deep CNN structure consisting of 16 layers with repeatable 3×3 convolution and max-pooling blocks. VGG16 is well-established in medical imaging research and serves as a strong, interpretable starting point.

To determine the most effective transfer learning strategy, various freeze rates of 0.01, 0.05, 0.10, 0.20, 0.50, and 0.75 were considered, and the following formula was used to calculate how many layers of the pretrained base model to freeze: num_freeze_layer = int(len(base_model.layers) × freeze_rate).

The freeze rate controls how much of the original model’s learned features are retained versus fine-tuned on the new task. In general, higher freeze rates, such as 0.50 or 0.75, are preferable when working with small datasets or datasets like the original training data (ImageNet), as they help prevent overfitting and preserve general visual features. Conversely, lower freeze rates, such as 0.01 or 0.05, are more suitable for large or highly domain-specific datasets, where extensive fine-tuning is necessary. For many practical applications, mid-range freeze rates like 0.10 or 0.20 often provide the best balance, allowing the model to adapt to new data while still leveraging pretrained knowledge effectively.

Most layers of the pretrained model were frozen, except for selected unfrozen layers, enabling selective fine-tuning to adapt high-level features to the target domain while preserving learned representations.

As part of the model architecture, a GlobalAveragePooling2D layer was incorporated after the convolutional base. This layer plays a crucial role in reducing the spatial dimensions of the feature maps while preserving the most important information. Unlike traditional flattening, which converts the entire feature map into a long vector (often leading to many parameters), GlobalAveragePooling2D computes the average of each feature map, resulting in a much more compact representation. This not only reduces the risk of overfitting but also maintains the model’s spatial awareness and generalization ability. Additionally, it helps bridge the convolutional layers and the dense output layer in a more efficient and scalable way, especially when working with transfer learning models.

To further mitigate overfitting and improve generalization, a Dropout layer was added after the GlobalAveragePooling2D layer. Dropout works by randomly setting a fraction of the input units to zero during training, which prevents the model from becoming too reliant on specific neurons. Several dropout rates (0.2, 0.3, 0.4, and 0.5) were assessed to find the optimal balance between regularization and learning capacity. Lower dropout rates like 0.2 provided lighter regularization and allowed the model to retain more features, while higher rates like 0.5 offered stronger regularization but at the cost of slower learning. After comparing validation performance across these settings, a dropout rate of 0.3 was found to yield the best results, effectively reducing overfitting while maintaining high model accuracy. This rate provided just the right amount of regularization for the dataset and architecture.

Although the input dataset was prenormalized, a BatchNormalization layer was still incorporated within the model architecture. While input normalization standardizes the data fed into the model, BatchNormalization operates between layers, dynamically normalizing the activations during training. This helps address internal covariate shift, where the distribution of layer inputs changes due to updates in earlier layers, thus stabilizing training, enabling higher learning rates, and often improving generalization. Even with normalized input data, this internal normalization contributed to faster convergence and improved validation performance across experiments.

To determine the ideal size for the fully connected (dense) layer, various unit sizes (32, 64, 128, 256, and 512) were assessed. The number of units in the dense layer directly impacts the model’s ability to learn complex patterns. Smaller sizes like 32 or 64 limit the model’s capacity and are often suitable for simpler tasks or small datasets. Larger sizes like 256 or 512 increase representational power but also introduce a greater risk of overfitting, especially if the dataset is not sufficiently large or diverse. It was observed that as the number of units increased, the model’s ability to capture nuanced patterns improved up to a point. Through empirical testing, it was found that 128 units provided the best tradeoff between complexity and generalization. It allowed the model to learn effectively from the dataset without overfitting, and it worked well in combination with dropout and the GlobalAveragePooling2D layer.

To assess the real-time applicability of our target system, 2 model architectures were compared to balance performance and efficiency. Both began with a pretrained base model, followed by GlobalAveragePooling2D, BatchNormalization, and an initial Dropout and Dense layer. The first architecture included an additional Dropout and Dense layer, designed to improve representational capacity and regularization. The second architecture was more streamlined, using only a single Dropout and Dense layer before the output.

In the context of real-time deployment, model efficiency is crucial. While the deeper architecture offered slightly better training performance, it came at the cost of increased latency and model complexity. Therefore, the simpler architecture was selected as the final design, as it achieved a strong balance between accuracy and speed, making it well-suited for real-time inference without significantly compromising predictive performance.

The Dense layer had rectified linear unit activation, He-normal initialization, and L2 regularization. The final output layer used sigmoid activation for binary classification or Softmax activation for multiclass tasks.

As part of the optimization strategy, several well-known optimizers, including SGD, RMSprop, Adam, Nadam, and AdamW, were evaluated. Each optimizer has unique strengths: SGD offers strong theoretical foundations but typically requires fine-tuned hyperparameters; RMSprop is effective in handling nonstationary objectives; Adam combines momentum and adaptive learning rates, leading to fast convergence; and Nadam incorporates Nesterov momentum into Adam for smoother updates. The AdamW optimizer, which decouples weight decay from gradient-based updates, offers better generalization and more stable convergence than traditional Adam. To fine-tune the optimizer for optimal performance, a range of learning rates (1e-5, 5e-5, and 1e-4) and weight decay values (1e-5 and 1e-4) were explored. This tuning allowed the model to adapt effectively to the complexity of the dataset while minimizing overfitting. After extensive experimentation, it was found that a learning rate of 5e-5 combined with a weight decay of 1e-5 yielded the best results, providing smooth convergence, strong validation accuracy, and robust generalization. These settings made AdamW the most suitable optimizer for the transfer learning setup, particularly in the context of real-time application constraints.

Binary cross-entropy was used as the loss function for binary classification, while categorical cross-entropy was employed for multiclass settings. Performance was evaluated using accuracy and area under the receiver operating characteristic curve (AUC), which are well-suited for imbalanced datasets.

To enhance training efficiency and prevent overfitting, several callbacks were incorporated. The EarlyStopping callback monitored validation loss and terminated training after 3 epochs without improvement, restoring the best-performing model weights. ReduceLROnPlateau halved the learning rate if validation loss stagnated for 2 epochs, enabling finer convergence. A model checkpointing strategy saved the full model, including weights and architecture, to a specified directory at each epoch, regardless of validation performance, ensuring training continuity and recovery if interrupted.

#### Hyperparameter Tuning

Automated hyperparameter optimization was performed using the Hyperband algorithm implemented in Keras Tuner. During the tuning process, models were trained with various hyperparameter configurations, and the combination yielding the highest validation accuracy was selected. Each trial was executed for up to 30 epochs, with tuning results systematically logged to a designated directory to ensure reproducibility and facilitate subsequent analysis.

The tuning process was orchestrated by a centralized function that built the model based on sampled hyperparameters, applied callbacks, conducted training on the training and validation splits, and identified the best-performing configuration. The final model, constructed using this optimal configuration, was retrained on the full training data and saved for future deployment or evaluation.

#### Advantages of the Framework

This framework offers several key advantages. It automates the search for critical hyperparameters, such as dropout rates, dense layer sizes, and learning rates, reducing the reliance on manual tuning. Leveraging pretrained models improves learning efficiency and generalization, which is particularly valuable when working with small or noisy medical datasets. Furthermore, the integration of early stopping, adaptive learning rate scheduling, and model checkpointing ensures robust, reliable training. Collectively, these strategies contribute to the development of accurate and generalizable deep learning models suitable for real-world clinical applications.

### Model Evaluation

To assess the generalization performance of each trained model, a comprehensive evaluation was conducted using a separate, unseen test dataset. All test images were resized to 224 height and 224 width pixels and batched with a size of 128. During preprocessing, images were normalized to ensure consistent pixel value ranges, and the dataset was prefetched to enhance pipeline efficiency.

To evaluate deep learning architectures for COVID-19 detection, a variety of models from different families were selected. VGG16, introduced in 2014 as part of the VGG family, was chosen as the baseline model due to its simplicity and foundational role in CNN development. It achieved 71.3% top 1 accuracy with 138 million parameters and 41 layers. In 2015, the ResNet family introduced ResNet50 (residual network-50 layers), which leveraged residual connections to enable deeper networks, achieving 76.2% accuracy with 25.6 million parameters and 177 layers. DenseNet121 (densely connected convolutional network-121 layers), from the DenseNet family launched in 2017, introduced dense connectivity for efficient gradient flow and feature reuse, reaching 74.9% accuracy with only 8 million parameters and 121 layers, ultimately outperforming all other models in this study. The MobileNet family (2017‐2019) contributed MobileNetV2, optimized for mobile devices using inverted residuals, with 71.8% accuracy, 3.4 million parameters, and 88 layers. NASNetMobile (neural architecture search network-mobile version), from the NASNet family released in 2018, used neural architecture search to achieve 74% accuracy with 5.3 million parameters and 88 layers. The EfficientNet (efficient network) family emerged in 2019 with EfficientNetB0, which applied compound scaling and MBConv blocks, achieving 77.1% accuracy with 5.3 million parameters and 237 layers. Its successor, EfficientNetV2B0, released in 2021, improved training speed and accuracy, delivering 78.1% accuracy with 7.1 million parameters and 329 layers. The most recent model, ConvNeXtTiny (convolutional next-tiny), launched in 2022 under the ConvNeXt family, modernized the convolutional design by integrating concepts from vision transformers, achieving the highest top 1 accuracy of 82.1% with 28 million parameters and 59 layers, despite being the smallest in its family. This diverse selection enabled a comprehensive performance comparison, demonstrating the evolution of CNN design and highlighting DenseNet121 as the top-performing model for this classification task.

Each trained model, beginning with VGG16 and followed by ConvNeXtTiny, ResNet50, EfficientNetB0, EfficientNetV2B0, DenseNet121, MobileNet, MobileNetV2, and NASNetMobile, was individually loaded and evaluated. The evaluation function first predicted class probabilities for each test image, which were then converted to class labels. For binary classification tasks, a threshold of 0.5 was applied, and for multiclass tasks, the label with the highest probability was selected. Ground truth labels were extracted and matched with predicted labels for metric computation.

The following performance metrics were used for evaluation: accuracy, precision, recall, *F*_1_-score, and AUC. Depending on the number of classes in the dataset, macro or binary averaging was automatically selected for precision, recall, and *F*_1_-score. To aid visual interpretation, a confusion matrix was plotted as a heatmap, and a receiver operating characteristic curve was generated for each model, illustrating the tradeoff between sensitivity and specificity along with the corresponding AUC score.

Performance metrics for each model were stored in a centralized results dictionary, enabling straightforward comparison. Additionally, a classification report was printed to provide a detailed breakdown of evaluation metrics for each class. Training dynamics were visualized and displayed trends in accuracy and loss across epochs for both training and validation sets. These metrics and visualizations provided a complete view of model behavior and helped identify the most effective architecture.

### Definitions of Evaluation Metrics

#### Accuracy

The formula for accuracy is as follows: accuracy = (true positive + true negative) / (true positive + true negative + false positive + false negative). Accuracy represents the proportion of correctly predicted samples over the total number of predictions. It is a suitable metric when the dataset is balanced across classes.

#### Precision (Positive Predictive Value)

The formula for precision is as follows: precision = (true positive) / (true positive + false positive). Precision measures the correctness of positive predictions. It is especially important when the cost of false positives is high.

#### Recall (Sensitivity or True Positive Rate)

The formula for recall is as follows: recall = (true positive) / (true positive + false negative). Recall assesses the model’s ability to identify actual positives. It is critical in scenarios like medical diagnosis, where missing positive cases can have serious consequences.

#### *F*_1_-Score (Harmonic Mean of Precision and Recall)

The formula for *F*_1_-score is as follows: *F*_1_-score = 2 × ([precision × recall] / [precision + recall]). The *F*_1_-score balances precision and recall and is particularly useful when working with imbalanced datasets.

#### AUC Metric

The receiver operating characteristic curve plots the true positive rate (recall) against the false positive rate. The AUC represents the probability that a randomly chosen positive instance is ranked higher than a randomly chosen negative instance. A higher AUC indicates better model discrimination capability.

### Implementation

The proposed method was implemented in Python using Keras, a high-level neural network application programming interface (API) built on top of the TensorFlow framework. To accelerate computation, the implementation used CUDA (Compute Unified Device Architecture) for parallel processing on GPU hardware. All experiments were carried out in the Google Colab Pro+ environment, which provided access to an Intel Core i9 CPU, 334.6 GB of RAM, an NVIDIA v2-8 TPU, and 225.3 GB of disk storage. The full implementation, along with the pretrained models, is publicly available on GitHub [32] to support reproducibility and further research.

### Ethical Considerations

This study did not involve the recruitment of human participants or the collection of new patient data; therefore, institutional review board or research ethics board approval was not required. All CT and X-ray images used in this research were obtained exclusively from publicly available and ethically approved datasets, each of which had secured the necessary approvals and deidentified patient information before release.

As the data were fully anonymized and publicly accessible, informed consent from individual patients was not applicable. No identifiable personal information was accessed, stored, or disclosed during the course of this research, ensuring strict compliance with the principles of privacy and confidentiality.

No financial or nonfinancial compensation was provided to patients or data contributors, as all datasets were obtained from open-access repositories made available for scientific and educational purposes.

## Results

### Hypothesis-Driven Evaluation

#### High Accuracy Across Variants

The curated dataset, representing emerging variants, such as Pi, Rho, Xec, and JN.1, enabled model training and validation with high precision.

#### Performance Versus Traditional Tests

The deep learning model outperformed traditional tests in sensitivity for variant cases. For instance, while RT-PCR sensitivity dropped for Pi and JN.1, the model maintained >98% recall in cross-validation trials.

#### Generalizability

By incorporating images from 19 countries across different imaging modalities and population groups, the model exhibited stable performance across validation subsets with different geographic and demographic characteristics.

#### Differentiation From Other Pneumonias

Fine-grained classification enabled the model to distinguish COVID-19 pneumonia from other respiratory infections (bacterial and atypical pneumonias), achieving a specificity of 96.9% and an *F*_1_-score of 97.9%.

### Data Collection

To build a generalizable and robust deep learning model for COVID-19 pneumonia diagnosis, a diverse, multi-institutional imaging dataset combining both CT and X-ray modalities was curated. The dataset features a total of 87,231 patients, including 59,961 COVID-19–positive cases and 27,270 normal or non-COVID pneumonia cases, with an age range of 0 to 100 years, gender groups of male and female, representation of 19 countries, and imaging modalities comprising chest CT scans and chest X-rays.

#### Data Collection Summary

A diverse set of imaging datasets spanning CT and X-ray modalities was compiled from multiple countries to ensure model generalizability and robustness. A total of 87,231 images were identified. The largest contributor was BIMCV-COVID19 from Spain with 79,023 (90.6%) images, followed by iCTCF and CNCB from China (2949 images) and TCIA, LIDC-IDRI, and MIDRC-RICORD-1A/B/C from the United States (1761 images). Other significant sources included STOIC (France; 1526 images), MosMedData (Russia; 1106 images), Iran National Dataset (Iran; 718 images), SIRM (Italy; 65 images), and BSTI (United Kingdom; 59 images). Additionally, radiological images were extracted from global resources like Radiopaedia and contributions from 11 other countries, each providing 24 cases (Multimedia Appendix 2). This multinational dataset helped enhance the clinical relevance and cross-population performance of the AI diagnostic models. BIMCV-COVID19 (Spain) contributed the largest number of both positive and negative samples, and there were smaller contributions from datasets such as SIRM (Italy), CHQC (China), and MIDRC-RICORD (United States) (Multimedia Appendix 3). The distribution highlights the dataset’s diversity and the class balance achieved across sources, which are critical for training robust and unbiased diagnostic models.

#### Imbalance Observation

Most data were collected from the BIMCV-COVID19 dataset (Spain), which, while enhancing the dataset’s size and regional representation, introduces a notable class imbalance. Specifically, COVID-19 positive cases (59,961) substantially outnumbered normal and non-COVID pneumonia cases (27,270). This disproportion primarily stems from the emphasis of public datasets on rapid COVID-specific data collection during the pandemic, which may skew model learning and diagnostic performance if not addressed.

### Data Preprocessing

To ensure robust model performance across varying demographics, modalities, and clinical conditions, a comprehensive data preprocessing pipeline was applied. The steps undertaken effectively addressed initial issues of class imbalance, missing metadata, and image inconsistencies.

#### Class and Source Balancing

After applying undersampling and dropping countries with ultra-low samples, 3000 cases from Spain, 2949 from China, 1761 from the United States, 1526 from France, 1106 from Russia, and 718 from Iran were retained, with 7572 COVID cases and 3488 non-COVID cases.

#### Metadata Imputation Results

Age had 5537 missing values imputed using country-wise medians, and gender had 5511 missing values imputed using country-wise modes. Metadata completeness improved to 100%, allowing demographic-aware stratification and analysis during model evaluation.

#### Age and Gender Distribution

The age range was 0 to 100 years. After removing outliers, the age group distribution remained imbalanced, with adults (n=7219) forming the majority, followed by elderly (n=2234), young adults (n=1553), and children (n=54). The distribution reflects a population skew that may influence age-specific modeling outcomes, and stratified labels by age group ensure balanced data. Gender balance included 34.1% (3398/9954) males and 65.9% (6556/9954) females. After processing, the dataset had 4509 positive and 2047 negative cases among females, and 2307 positive and 1091 negative cases among males. This balanced demographic composition supports robust model evaluation across diverse patient profiles.

#### Dataset Overview After Balancing

After applying country-based filtering, undersampling, and augmentation, a more equitable distribution of samples across countries and classes was achieved. The total number of curated images was 11,052, with 8842 images in the training set and 2210 images in the validation set. Image dimensions were resized to 75×75 pixels with 3 RGB channels, and normalization was applied with all pixel values rescaled to the [0, 1] range.

#### Country-Level Balance (Postaugmentation)

Augmentation techniques were applied particularly to underrepresented classes to reduce class imbalance and enhance model generalization. A balanced representation (2034 COVID samples per country) was achieved across 6 key contributors (China, France, Iran, Russia, Spain, and the United States). Similarly, normal samples were balanced at 1249 images across the same regions, improving generalization across populations (Multimedia Appendix 4).

The dataset comprised 12,204 COVID-19–positive images and 7494 normal images, indicating a moderate class imbalance favoring positive cases. This distribution highlights the need for balancing techniques such as augmentation during model training.

#### Augmentation Impact

The applied augmentation techniques (flip, rotate, zoom, contrast, and translation) not only balanced the dataset but also increased image variability, simulating real-world noise and improving model resilience to unseen data (Multimedia Appendix 5). There was a nearly equal number of images per label (nearly 2000 per class) in each country, demonstrating successful class balancing to mitigate bias during model training.

#### Class Distribution (Postaugmentation)

There was an equal number of COVID-positive and normal (COVID-negative) images (12,204 each), reflecting the successful application of augmentation techniques to balance the dataset and prevent model bias due to class imbalance. Class distribution after augmentation is presented in Multimedia Appendix 6.

### Modeling

To ensure a fair and consistent evaluation, all models were trained using standardized input settings. Each image was resized to 224×224 pixels, producing an input shape of (224, 224, 3) to accommodate RGB color channels. Although the images originated in RGB format, they were converted to grayscale during preprocessing and normalized to a range of [0, 1] for efficient convergence.

All transfer learning architectures were trained for 30 epochs, a setting chosen to balance computational efficiency with sufficient learning. A batch size of 128 was used to maintain stable updates across mini-batches. Additionally, a shuffle buffer size of 10,000 ensured randomness in the training data pipeline, reducing overfitting risks.

This consistent training configuration was applied across all models (VGG16, ConvNeXtTiny, ResNet50, EfficientNetB0, EfficientNetV2B0, DenseNet121, MobileNet, MobileNetV2, and NASNetMobile).

Through hyperparameter tuning, the DenseNet121 architecture was found to yield the best performance. Its final configuration included dropout layer 1 with 0.3, dense layer 1 with 128 units, a learning rate of 0.00037758, and a weight decay of 7.4855e-05. This architecture and training regime were optimized to prevent overfitting while maintaining high model generalization on unseen data.

### Model Evaluation

**Figure 1.**
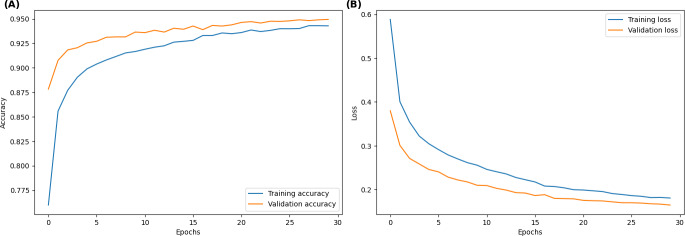
The training and validation (A) accuracy and (B) loss curves of DenseNet121 (densely connected convolutional network-121 layers) over 30 epochs, showing strong learning convergence with minimal divergence between the training and validation sets, which is an indicator of effective generalization.

Among the evaluated models, DenseNet121 delivered the best overall performance, achieving 98% accuracy, 96.8% precision, 98.8% recall, and an AUC of 0.998, indicating a well-balanced and highly effective binary classifier (Figure 1; Table 4). NASNetMobile and VGG16 also showed strong performance, with high scores across all metrics, making them solid alternatives. ResNet50 showed competitive results but fell slightly short of the top 3 models, particularly in precision. On the other hand, models, such as EfficientNetB0, EfficientNetV2B0, ConvNeXtTiny, and MobileNet, showed poor performance. Despite their perfect recall, their low precision and AUC values suggest that they overpredicted the positive class, leading to high false positive rates. MobileNetV2, despite a decent accuracy and AUC, failed to maintain balance across precision and recall, making it less suitable for reliable classification in this context. Given its superior and consistent results, DenseNet121 stands out as the most suitable model for deployment, offering both robustness and high predictive accuracy for this binary classification task.

**Table 4.** Comparative analysis findings of performance metrics for all transfer learning models applied to the task of COVID-19 detection from medical images.

Model	Accuracy	Precision	Recall	*F*_1_-score	AUC^a^
EfficientNet^b^B0	0.46219	0.46219	1.00000	0.63218	0.33122
EfficientNetV2B0	0.46219	0.46219	1.00000	0.63218	0.63435
MobileNet^c^	0.54306	0.50287	0.99545	0.66819	0.93267
ConvNeXtTiny^d^	0.46219	0.46219	1.00000	0.63218	0.50726
ResNet50^e^	0.92542	0.87885	0.97273	0.92341	0.99033
VGG16^f^	0.93487	0.91087	0.95227	0.93111	0.98431
NASNetMobile^g^	0.95798	0.93290	0.97954	0.95565	0.99619
MobileNetV2	0.97370	0.96874	0.97773	0.97321	0.97990
DenseNet121^h^	0.98004	0.96882	0.98864	0.97863	0.99830

a AUC: area under the receiver operating characteristic curve.

b EfficientNet: efficient network.

c MobileNet: mobile network.

d ConvNeXtTiny: convolutional next-tiny.

e ResNet50: residual network-50 layers.

f VGG16: Visual Geometry Group network-16 layers.

g NASNetMobile: neural architecture search network-mobile version.

h DenseNet121: densely connected convolutional network-121 layers.

**Figure 2.**
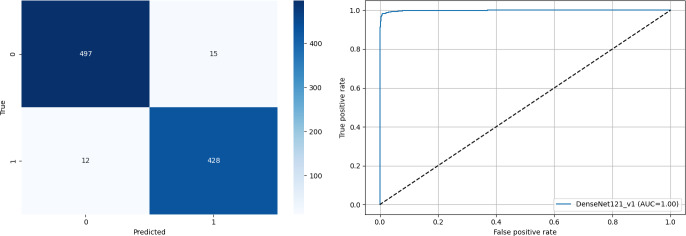
(A) Confusion matrix and (B) receiver operating characteristic curve for DenseNet121 (densely connected convolutional network-121 layers). AUC: area under the receiver operating characteristic curve.

The confusion matrix reflects DenseNet121’s exceptional classification accuracy with minimal misclassification (Figure 2A).

The balance indicates that the model is not only highly accurate but also well-calibrated in terms of sensitivity (recall) and specificity.

The receiver operating characteristic curve further supports these results, with an AUC of 1.00, demonstrating near-perfect separation between positive and negative classes. The curve closely hugs the top-left corner, indicating an excellent tradeoff between the true positive rate and false positive rate (Figure 2B).

Together, these visualizations affirm DenseNet121’s reliability and robustness for the binary classification task of COVID-19 detection, outperforming other evaluated architectures in both quantitative metrics and qualitative visual assessment.

## Discussion

### Summary

The results show that DenseNet121 achieved the highest performance, with 98% accuracy, 96.8% precision, and 98.8% recall, demonstrating robust diagnostic capabilities.

### Conclusion

This study introduces a robust deep learning framework for COVID-19 diagnosis using chest X-ray and CT imaging, emphasizing both high model performance and real-world deployment feasibility. Leveraging imaging data from 19 countries across diverse age groups, genders, and COVID-19 variants, the study used comprehensive preprocessing, undersampling, and data augmentation techniques to ensure balanced and representative datasets. To ensure practical deployment, models were optimized through quantization and pruning, making them lightweight and suitable for web-based diagnostic platforms via cloud APIs (Flask or RESTAPI with TensorFlow Serving) and mobile apps using TensorFlow Lite or ONNX for on-device diagnosis, which can be especially valuable in low-resource and rural settings. The framework further integrates Grad-CAM visualizations for explainability, federated learning for privacy-preserving collaboration across hospitals, and longitudinal monitoring for tracking long COVID or reinfection cases. These features collectively position the system as a clinically relevant, mutation-resilient, and scalable solution for COVID-19 screening and triage in modern health care environments. For future work, there is an aim to extend this framework to multiclass classification, distinguishing between lung pathologies such as tuberculosis, AIDS, and COVID-19. This initiative will be pursued in collaboration with clinicians to enhance diagnostic specificity and clinical utility.

### Future Work

#### Clinical Validation Across Institutions

There is an aim to collaborate with multiple hospitals and diagnostic centers to externally validate the model on institution-specific datasets. This will help assess the model’s generalizability and robustness across different scanners, protocols, and patient populations.

#### Integration With EHRs

Work is underway to integrate the diagnostic tool with EHR systems for seamless access to patient history and real-time imaging data, enabling context-aware predictions and decision support.

#### Deployment on Web and Mobile Platforms

The final model is being optimized using techniques, such as quantization and pruning, for deployment on edge devices and cloud platforms. This will support real-time diagnosis via a web interface and mobile app, particularly in resource-constrained or rural areas.

#### Regulatory Readiness and Clinical Trials

Documentation and performance benchmarks are being prepared to pursue regulatory approval (Conformité Européenne marking and Food and Drug Administration clearance). A prospective clinical trial is also being designed to measure diagnostic impact in a real-world setting.

#### Extension to Long COVID and Follow-Up Monitoring

There is a plan to adapt the system for longitudinal analysis, enabling clinicians to track radiological changes over time, which can be useful for monitoring long COVID progression or reinfections.

#### Federated Learning for Privacy-Preserving AI

To support data privacy and multi-institutional collaboration, an attempt will be made to explore federated learning frameworks that allow model training on decentralized data without sharing patient images.

## Supplementary material

10.2196/75015 Multimedia Appendix 1 Distribution of COVID-positive and normal chest images by country.

10.2196/75015 Multimedia Appendix 2 Source-wise distribution of imaging data used in the study.

10.2196/75015 Multimedia Appendix 3 Label-wise distribution of COVID-positive and negative cases across various data sources.

10.2196/75015 Multimedia Appendix 4 Bar chart of image count per label and country after data augmentation, illustrating a balanced distribution of COVID and normal images across 6 countries, which ensured class uniformity for training deep learning models.

10.2196/75015 Multimedia Appendix 5 Bar chart of image count per label and country, showing the distribution of COVID-19 and normal images across 6 countries after data augmentation.

10.2196/75015 Multimedia Appendix 6 Bar chart of the total image count per label after augmentation.

## Abbreviations

AI: artificial intelligence
API: application programming interface
AUC: area under the receiver operating characteristic curve
BIMCV-COVID19: Banco de Imágenes Médicas de la Comunidad Valenciana–COVID-19
CNCB: China National Center for Bioinformation
CNN: convolutional neural network
ConvNeXtTiny: convolutional next-tiny
CT: computed tomography
DenseNet121: densely connected convolutional network-121 layers
EfficientNet: efficient network
EHR: electronic health record
iCTCF: CT images and clinical features for COVID-19
LFD: lateral flow device
LIDC-IDRI: Lung Image Database Consortium image collection
MIDRC-RICORD: Medical Imaging Data Resource Center - RSNA International COVID-19 Open Radiology Database
MobileNet: mobile network
NASNetMobile: neural architecture search network-mobile version
RAT: rapid antigen test
RestNet50: residual network-50 layers
RT-PCR: reverse transcription–quantitative polymerase chain reaction
SIRM: Società Italiana di Radiologia Medica e Interventistica
STOIC: Study of Thoracic CT in COVID-19
TCIA: The Cancer Imaging Archive
VGG16: Visual Geometry Group network-16 layers
WHO: World Health Organization

## References

[1] (2020). Pneumonia of unknown cause – China. World Health Organization.

[2] Coronavirus disease (COVID-19) - Overview. World Health Organization.

[3] (2025). COVID-19: new variants in 2025. Ada Health.

[4] (2023). Weekly epidemiological update on COVID-19 - 25 August 2023. World Health Organization.

[5] (2020). COVID-19 Public Health Emergency of International Concern (PHEIC) Global research and innovation forum. World Health Organization.

[6] (2025). COVID-19 - global situation. World Health Organization.

[7] Katella K (2024). 3 things to know about XEC, the dominant COVID strain. Yale Medicine.

[8] (2025). Surveillance and data analytics. Centers for Disease Control and Prevention.

[9] (2025). WHO’s 2025 updates on COVID-19 variants: focus on XEC, testing, and recovery. ASSURE.

[10] (2025). Tracking SARS-CoV-2 variants. World Health Organization.

[11] Coronavirus disease (COVID-19) - Symptoms. World Health Organization.

[12] (2025). COVID-19 symptoms: Omicron vs. Delta. Ada Health.

[13] Ullah SMA, Islam MM, Mahmud S, Nooruddin S, Raju S, Haque MR (2021). Scalable telehealth services to combat novel coronavirus (COVID-19) pandemic. SN Comput Sci.

[14] Wang L, Lin ZQ, Wong A (2020). COVID-Net: a tailored deep convolutional neural network design for detection of COVID-19 cases from chest x-ray images. Sci Rep.

[15] Reshan MSA, Gill KS, Anand V (2023). Detection of pneumonia from chest x-ray images utilizing MobileNet model. Healthcare (Basel).

[16] Mujahid M, Rustam F, Álvarez R, Luis Vidal Mazón J, Díez I de la T, Ashraf I (2022). Pneumonia classification from x-ray images with Inception-V3 and Convolutional Neural Network. Diagnostics (Basel).

[17] Ghaderzadeh M, Asadi F, Jafari R, Bashash D, Abolghasemi H, Aria M (2021). Deep convolutional neural network-based computer-aided detection system for COVID-19 using multiple lung scans: design and implementation study. J Med Internet Res.

[18] Jiang W, Ji W, Zhang Y (2022). An update on detection technologies for SARS-CoV-2 variants of concern. Viruses.

[19] Miró Catalina Q, Fuster-Casanovas A, Solé-Casals J, Vidal-Alaball J (2022). Developing an artificial intelligence model for reading chest x-rays: protocol for a prospective validation study. JMIR Res Protoc.

[20] Wang Q, Mellis IA, Ho J (2024). Recurrent SARS-CoV-2 spike mutations confer growth advantages to select JN.1 sublineages. Emerg Microbes Infect.

[21] Scott Mader K (2021). The Lung Image Database Consortium image collection (LIDC-IDRI). IEEE DataPort.

[22] SIRM - Società Italiana di Radiologia Medica e Interventistica.

[23] (2023). BIMCV-COVID19, Conjuntos de datos relacionados con el curso de patología de COVID19 [Article in Spanish]. BIMCV.

[24] iCTCF: CT images and clinical features for COVID-19. National Genomics Data Center - China National Center for Bioinformation.

[25] CT images in COVID-19. The Cancer Imaging Archive.

[26] MIDRC-RICORD-1A. The Cancer Imaging Archive.

[27] MIDRC-RICORD-1B. The Cancer Imaging Archive.

[28] MIDRC-RICORD-1C. The Cancer Imaging Archive.

[29] STOIC2021 - COVID-19 AI Challenge. STOIC2021 Grand Challenge.

[30] COVID-19. Radiopaedia.

[31] Datasets [Article in Russian]. Center of Diagnostics and Telemedicine.

[32] Dharmik A (2025). COVID-19-APP. GitHub.

