# Supplementary figures and images for: COVID-19 Pneumonia Diagnosis Using Medical Images: Deep Learning–Based Transfer Learning Approach
Source: JMIRx Med. 2025 Sep 26;6:e75015. doi: 10.2196/75015 (PMC12468159; doi:10.2196/75015)

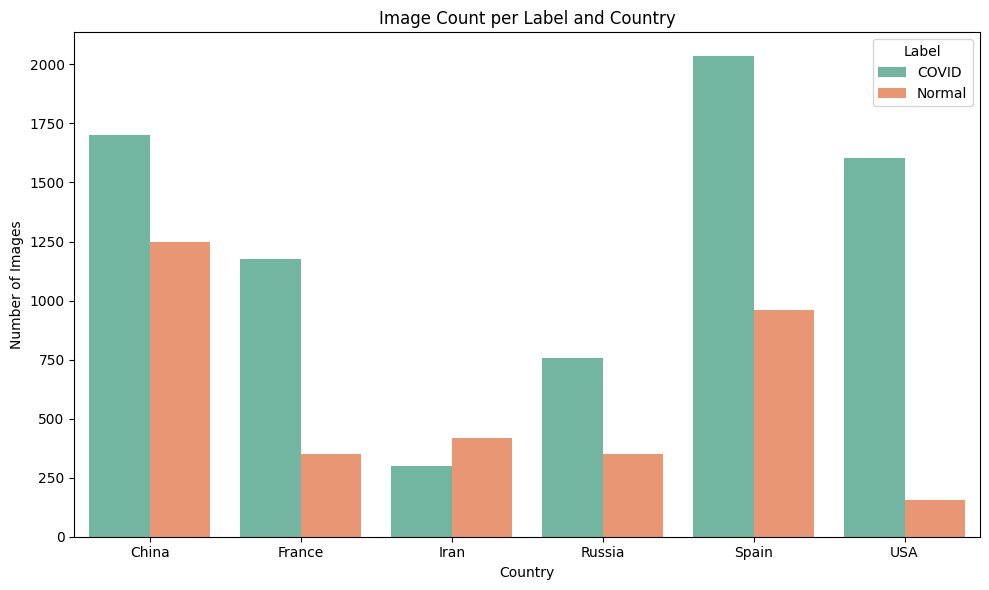

Supplement: Multimedia Appendix 1 [file xmed-v6-e75015-s001.png]

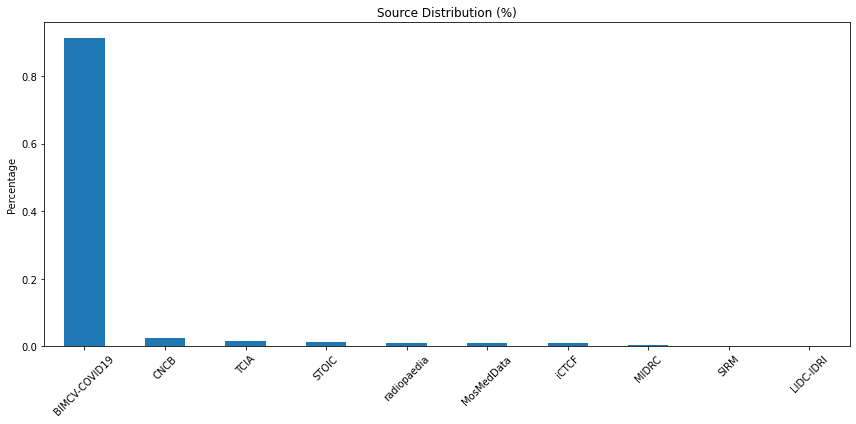

Supplement: Multimedia Appendix 2 [file xmed-v6-e75015-s002.png]

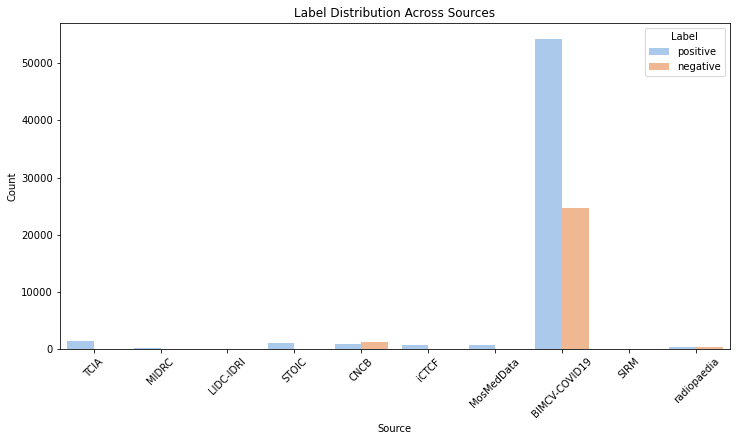

Supplement: Multimedia Appendix 3 [file xmed-v6-e75015-s003.png]

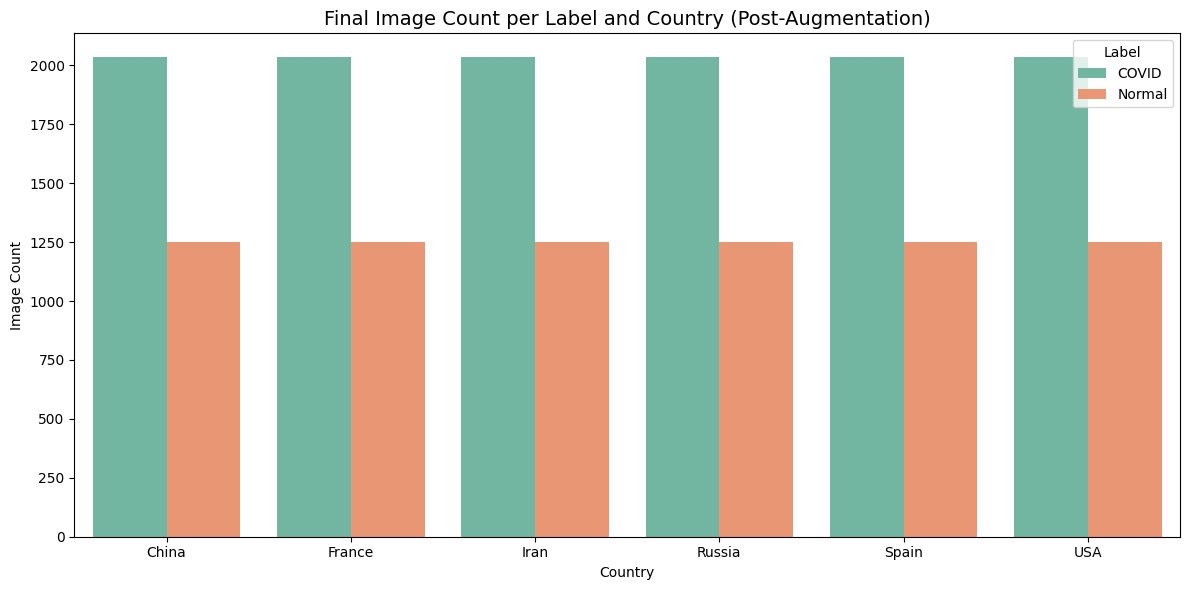

Supplement: Multimedia Appendix 4 [file xmed-v6-e75015-s004.png]

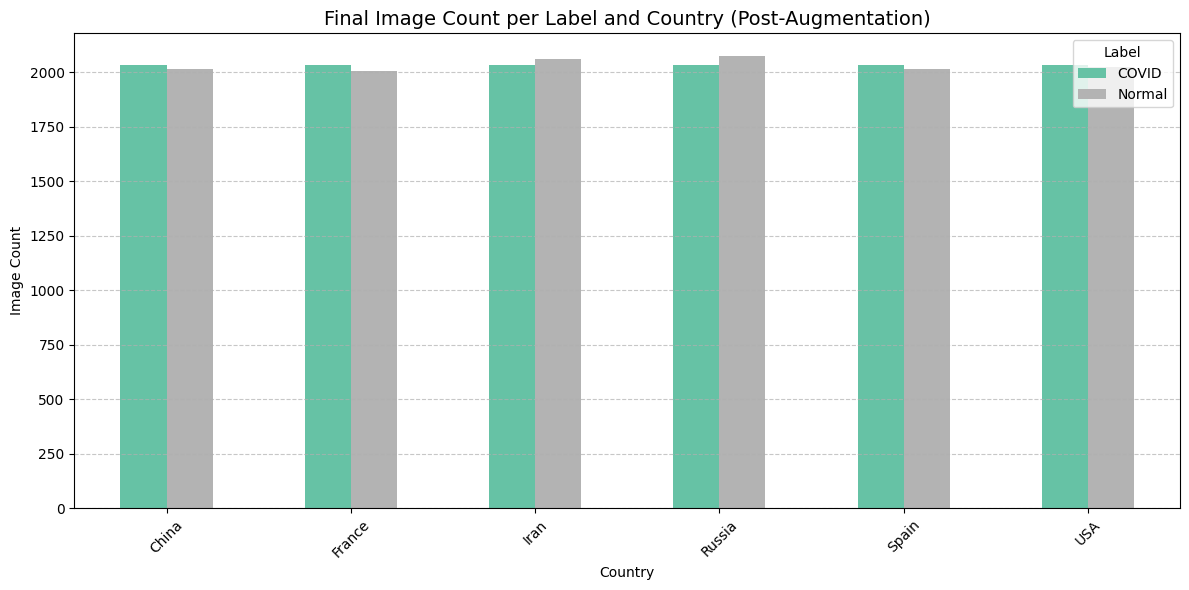

Supplement: Multimedia Appendix 5 [file xmed-v6-e75015-s005.png]

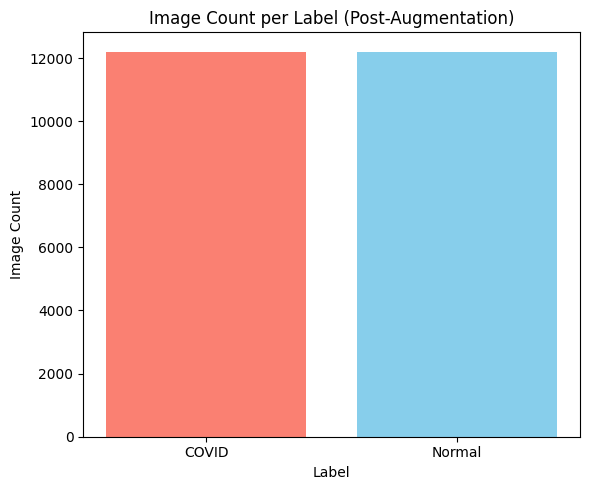

Supplement: Multimedia Appendix 6 [file xmed-v6-e75015-s006.png]
